# Urban Automation Networks: Current and Emerging Solutions for Sensed Data Collection and Actuation in Smart Cities

**DOI:** 10.3390/s150922874

**Published:** 2015-09-10

**Authors:** Carles Gomez, Josep Paradells

**Affiliations:** 1 Universitat Politècnica de Catalunya/Fundació i2Cat, C/Esteve Terradas, 7, Castelldefels 08860, Spain; 2 Universitat Politècnica de Catalunya/Fundació i2Cat, C/Jordi Girona, 1-3, Barcelona 08034, Spain; E-Mail: josep.paradells@entel.upc.edu

**Keywords:** smart cities, urban automation networks, sensor and actuator networks, low-power wireless networks, IEEE 802.15.4, IEEE 802.11, cellular networks, SIGFOX, DTN, Internet of Things

## Abstract

Urban Automation Networks (UANs) are being deployed worldwide in order to enable Smart City applications. Given the crucial role of UANs, as well as their diversity, it is critically important to assess their properties and trade-offs. This article introduces the requirements and challenges for UANs, characterizes the main current and emerging UAN paradigms, provides guidelines for their design and/or choice, and comparatively examines their performance in terms of a variety of parameters including coverage, power consumption, latency, standardization status and economic cost.

## 1. Introduction

During the last two centuries, the world population has been increasingly concentrating in urban areas. Today, around one half of all humans are living in cities, whereas the United Nations estimate that this figure will increase up to 75% by 2050. However, the current metropolitan growth model poses significant concerns in terms of environmental and economic sustainability [1]. Advances in a variety of technical fields offer the possibility to provide cities with *smart* mechanisms in order to allow efficient resource management and improved life quality for the citizen. In view of this opportunity, substantial efforts from municipalities, government agencies, the industry, standards development organizations and academia are being devoted to enable the *Smart City* [2,3,4,5,6,7,8,9,10,11].

In the last few years, numerous definitions of the Smart City term have been given [2,6,12]. However, as acknowledged by several authors in recent works, since the Smart City concept itself is developing, and because it involves actors from a variety of domains, a formal and widely accepted definition of Smart City does not exist yet [2,5,13]. Nevertheless, a commonly recognized, and in our opinion the crucial Smart City enabler is the use of Information and Communication Technologies (ICT) “to make the critical infrastructure components and services of a city more intelligent, interconnected, and efficient” [14].

Urban Automation Networks (UANs) are emerging as a central ICT component of the Smart City. UANs comprise fixed sensor (and/or actuator) nodes, backhauls and gateways that connect these nodes to core networks such as the Internet. Sensor nodes used in the Smart City are capable of detecting critical events and monitoring physical magnitudes relevant in the urban context. The collected information is transmitted to remote management centers where it can be processed and actions can be taken as a result, including the activation of urban actuators and publishing real-time or long-term information of interest to the citizen.

For the first time to our knowledge, this paper tackles the design, performance and economic cost of UANs under a comprehensive approach by: (i) introducing the UAN requirements, concept and architecture; (ii) presenting the main current and emerging UAN classes; (iii) evaluating their suitability for Smart City applications; and (iv) providing guidelines for UAN design and/or choice. The remainder of the paper is organized as follows. Section 2 illustrates UAN use cases and requirements. Section 3 describes the UAN generic architecture. Section 4 overviews the five main current and emerging UAN classes, which are discussed and evaluated in Section 5. Section 6 discusses the relationship between UANs and emerging mobile data collection networking paradigms in the Smart City context. Section 7 reviews Smart City modeling related work. The final section concludes the paper.

## 2. UAN Use Cases and Requirements

**Figure 1:**
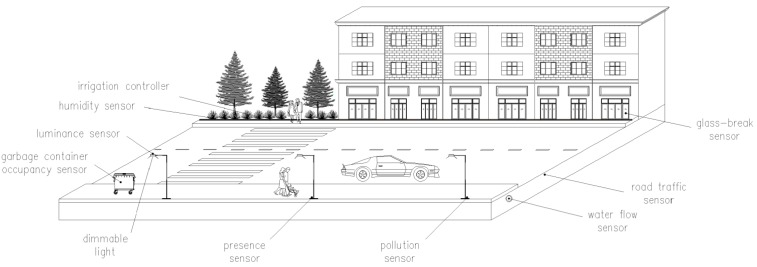
Example Urban Automation Networks (UAN)-enabled applications in a smart city.

UANs enable a wide spectrum of Smart City applications [5,6,8,11]. A list of relevant examples, along with their main features, is provided below and is illustrated in Figure 1.

- **Garbage collection**. Garbage containers can be provided with sensors that measure the containers’ occupancy. This information can be used to ensure compliance with recommendations on waste management, and to optimize garbage truck routes.
- **Lighting control**. Street light control can be automated based on measurements carried out by light sensors. Furthermore, street light intensity levels can be tuned based on the presence of people or vehicles, which can be detected by using presence sensors.
- **Green zone management**. Efficient and automated water irrigation systems can be applied in green zones by exploiting humidity sensors placed in the ground.
- **Environmental control**. Sensors can be used to monitor physical magnitudes relevant to the citizens’ and environmental health, such as weather conditions, air composition, acoustic pollution and ultraviolet solar radiation, among others.
- **Parking availability**. Several types of sensors (such as pressure, ultrasound or magnetic field sensors) may be used to identify empty parking spaces, which constitute a scarce resource in cities. The event of a parking space becoming available must be communicated quickly.
- **Street traffic**. Magnetic loops can be used for monitoring road traffic. The collected information can be published, in order to allow the citizens take suitable route decisions, avoid congested areas and minimize their contribution to air pollution. Furthermore, traffic lights may be intelligently controlled based on the current road traffic state.
- **Utility infrastructure**. Large equipment infrastructures from utility companies are deployed in the city, often underground, for providing gas, electricity, water, telecommunications and sewage services. Use of appropriate underground sensors can dramatically decrease failure detection times, help identify the location of breakdowns or leaks, and allow preventive maintenance.
- **Security**. Presence, proximity or even glass-break sensors may be used to detect or prevent intrusion into municipality areas (e.g., buildings, parks, *etc.*) during time periods in which access is not allowed.

Table 1 shows requirements and characteristics of the presented Smart City applications that must be met by the UANs supporting them. Periodic notifications from sensor nodes constitute the main source of data traffic. These notifications, which also serve as implicit network health messages, are not subject to real-time requirements. A subset of the applications tolerate infrequent sensor node connectivity opportunities (e.g., twice per day). Data traffic is asymmetric in UANs since the messages sent by sensor nodes towards the gateway outnumber the messages sent to actuators (or to sensor nodes, e.g., for management tasks). Applications that involve event-based traffic require permanent connectivity and relatively low delay (e.g., up to around ten seconds). Certain applications pose strict requirements on the sensor node location. In such cases, the UAN must be capable of providing adequate coverage in the intended sensor node locations.

**Table 1:** Requirements of Smart City applications enabled by UANs. The values shown in this table have been obtained considering Smart City application descriptions found in the literature [5,6,11], as well as our own experience in the design and deployment of Smart City pilots [8,15,16].

	Event-Based Alerts	Notification Periodicity	Actuators Involved	Sensor Node Location Accuracy
**Garbage collection**	No	1 h–24 h	No	High
**Lighting control**	Yes (if presence sensors are used)	30 min	Yes	Medium
**Green zone management**	No	1 h–24 h	Yes	Medium
**Environmental control**	No	1 h	No	Low
**Parking availability**	Yes	5 min	No	High
**Street traffic**	No	5 min	Yes (traffic light control and info. panels)	High/Medium
**Utility infrastructure**	Yes	12 h	No	High
**Security**	Yes	5 min	Yes (for alarm activation)	High/Medium

## 3. UAN Generic Architecture

Enabling Smart City applications requires the deployment of UANs that satisfy application requirements efficiently. UANs comprise sensor and actuator nodes, a backhaul and at least one gateway. The main characteristics of these components and their organization within a UAN are described next.

### 3.1. Sensor and Actuator Nodes

Sensor and actuator nodes are typically simple computing devices with sensing and/or actuation capabilities that exhibit significant constraints in terms of memory and processing power. Because in Smart Cities sensing nodes generally outnumber actuator nodes, in this paper we use the term *sensor nodes* for the sake of simplicity. For deployment flexibility and cost efficiency, sensor nodes are usually provided with wireless communication technologies. In urban scenarios, sensor nodes may not rely on mains power availability in their intended location, and therefore they commonly need to use batteries as their energy source, although a tendency towards exploiting energy harvesting solutions is gaining popularity. In order to allow multiyear lifetime for the sensor nodes without mains power, they must be in sleep mode by default and operate under low duty cycle regimes (either by waking up periodically or upon event detection).

### 3.2. Backhaul

In the UAN context, a backhaul is a wireless networking infrastructure which offers connectivity and data transport between sensor nodes and a gateway. UAN backhauls range from single-hop to multihop approaches. In the first case, the link between the sensor node and the gateway constitutes the backhaul. In the second one, the backhaul is composed of a set of backhaul nodes (with their corresponding links), which are in charge of relaying the data collected by or sent to sensor nodes. In order to enable long sensor node lifetime, most communication models assume that backhaul nodes are always prepared to receive messages from sensor nodes (*i.e.*, their radios are in receive mode by default), and thus backhaul nodes must not suffer energy consumption limitations.

### 3.3. Gateway

A UAN gateway is a device with multiple communication interfaces which interconnects UAN backhauls with a core network (e.g., an Intranet or the Internet), generally by means of Metropolitan Area Network (MAN) or Wide Area Network (WAN) technologies, such as fiber-optics, Digital Subscriber Line (DSL), 2.5G/3G/4G, Power Line Communication (PLC), Ethernet variants, *etc*. For the sake of service availability, the gateway is commonly required to be mains-powered. In order to provide high UAN reliability, it is recommendable that a given sensor node can reach more than one gateway.

## 4. UAN Classes

**Figure 2:**
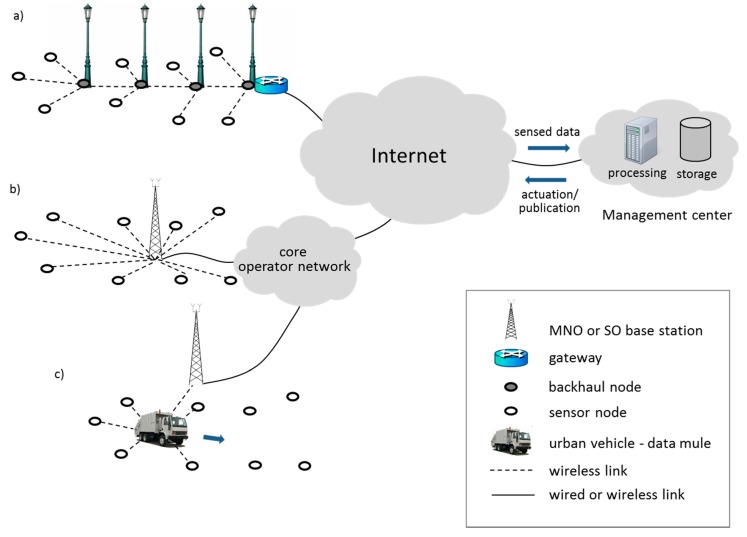
Architectures for sensed data collection and actuation in a smart city. (**a**) Architecture of Low-Rate (LR)-WPAN and Wireless LAN (WLAN) UANs; (**b**) Architecture of Mobile Network Operator (MNO) and SIM-less Operator (SO) UANs; (**c**) Architecture of Delay Tolerant Networking (DTN) UANs.

The UAN concept can be realized by following different approaches, which we categorize into UAN classes. In order to select the most suitable UAN class for a specific deployment, the requirements of the target applications and scenario must be considered. In fact, each UAN class has specific properties with crucial implications in terms of performance and economic cost. This section describes the five main current and emerging UAN classes, namely: Low-Rate Wireless Personal Area Network (LR-WPAN) UANs, Wireless LAN (WLAN) UANs, Mobile Network Operator (MNO) UANs, SIM-less Operator (SO) UANs and Delay Tolerant Networking (DTN) UANs. Figure 2 depicts their network architectures and how they integrate with the rest of smart city components.

### 4.1. LR-WPAN UANs

LR-WPAN UANs can be considered the quintessential UAN class, which is currently being deployed in many Smart City initiatives, and for which a majority of manufacturers and providers are offering equipment and solutions. In this UAN class, the sensor nodes use a variant of the IEEE 802.15.4 family [17] or exploit proprietary low-power wireless technologies at the physical and link layers of the protocol stack. IEEE 802.15.4 is the de facto radio interface used for low-power wireless applications. Amendments such as IEEE 802.15.4e and IEEE 802.15.4g provide optimizations that may be useful to overcome issues in urban scenarios such as multipath, narrowband interference, or fading due to obstacles [18,19].

In LR-WPAN UANs, the power consumption of the sensor nodes when their radios are in sleep mode is in the order of a few microwatt, whereas when the transceiver is active, nodes consume typically below one hundred milliwatt. The backhaul is an IEEE 802.15.4 multihop backbone composed of nodes that generally exhibit the same hardware characteristics as those of the sensor nodes. In addition, the backhaul nodes themselves can also be used for sensing. These nodes are always prepared to receive or forward data from or to the sensor nodes. The most straightforward solution for providing energy to the backhaul nodes is connecting them permanently to the mains power. To this end, a common solution is to install the backhaul nodes in streetlights. However, some lighting systems are only powered during nighttime. In this case, the backhaul nodes require load circuitry and rechargeable batteries in order to store energy for daytime operation.

**Figure 3:**
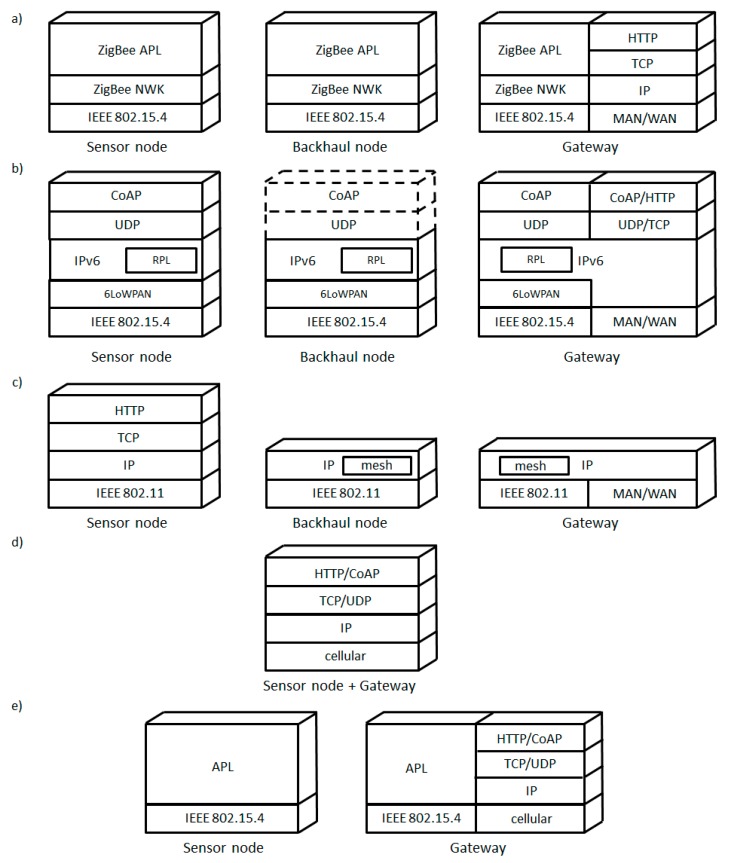
Protocol stacks and node types for different UAN classes. (**a**) LR-WPAN UAN based on ZigBee; (**b**) LR-WPAN UAN based on IP; (**c**) WLAN UAN; (**d**) MNO UAN; (**e**) DTN UAN. (Note: mesh functionality in WLAN UANs may be present at the link layer, as e.g., in IEEE 802.11s.).

There exist two main protocol architectures that are suitable for LR-WPAN UANs: ZigBee and the IP-based protocol stack for constrained node networks. In the first case, sensor and backhaul nodes implement the ZigBee protocol stack [20], which defines an application layer (APL) including commands and an end-to-end transport sublayer, and a network layer (NWK), which comprises routing and addressing functionality, on top of 802.15.4 (Figure 3a)). The gateway typically translates the ZigBee stack to an IP-based stack for Internet connectivity. The second LR-WPAN UAN type is based on the protocol suite developed by the IETF for constrained node networks (Figure 3b)). In this approach, sensor nodes use IPv6 since it has a vast address space and autoconfiguration capabilities. In order to enable IPv6 on top of IEEE 802.15.4, an adaptation layer called 6LoWPAN is introduced [21]. Multihop network connectivity is achieved by means of the RPL routing protocol [22]. Finally, an efficient binary protocol called CoAP is used, on top of UDP, at the application layer [23]. Backhaul nodes may support the whole protocol stack, although the CoAP/UDP protocols only need to be used if the backhaul nodes include sensing or management capabilities. CoAP has been designed to allow easy CoAP to HTTP message mapping, and thus the UAN gateway may effortlessly integrate with HTTP systems.

### 4.2. WLAN UANs

A WLAN UAN reuses existing IEEE 802.11 infrastructure already deployed in the city as the backhaul. In fact, many cities provide IEEE 802.11-based networks which offer several services to both the citizen and the municipality, such as Internet access, connectivity for surveillance systems, *etc.* Therefore, this UAN class requires the deployment of sensor nodes that must use a radio interface of the IEEE 802.11 family for compatibility with the backhaul.

Traditionally, 802.11 radios have been characterized by a relatively large power consumption. Nevertheless, in the last few years, so-called ultra-low-power WiFi modules have appeared in the market. In these modules, the power consumption in reception is comparable to that of LR-WPAN hardware, whereas the power consumption in transmission is greater than that of the latter (which is compensated by the fact that transmit times are shorter since greater data rates are used in the IEEE 802.11 family [24]).

The backhaul in WLAN UANs comprises nodes which may perform access point and mesh router functions. In this UAN type, a sensor node is in fact a client connected to one of the backhaul nodes. The latter have been deployed a priori (and thus are not equipped with sensors), usually in streetlights or traffic lights, and are provided with mains power.

In WLAN UANs, the sensor nodes implement a classic IP-based protocol stack over IEEE 802.11 (Figure 3c), which does not need the 6LoWPAN adaptation layer (although they would benefit in terms of energy savings from 6LoWPAN features such as header compression). Backhaul nodes may support mesh routing functionality at the network layer or at the link layer (e.g., by using IEEE 802.11s [25]). The gateway is an IP router, deployed to connect the existing WLAN infrastructure to the Internet.

### 4.3. MNO UANs

A third class of UANs is based on MNO cellular technology, which has traditionally been used for mobile voice and data communications, as the radio interface for sensor nodes in machine-to-machine (M2M) (In several circles, communication between machines by using cellular technologies has been denoted by M2M.) applications. In fact, the advent of the Short Message Service (SMS) allowed machines to carry out transactions through a cellular network. Later, 2.5G technologies, such as the General Packet Radio Service (GPRS), provided added value to M2M by allowing the use of IP, the lingua franca of current data networks. The performance enhancements offered by subsequent generations of cellular technologies, such as 3G or initial 4G variants are not particularly relevant for M2M, which typically involves short-sized and infrequent data exchanges. Instead, widespread coverage, simplicity, low power consumption and price are much more important attributes, for which 2G/2.5G provides currently the best solution [26]. However, future MNO UANs will benefit from enhancements specifically tailored to M2M communications (see this section).

In GPRS-based MNO UANs, in order to set the radio interface in active mode, sensor nodes have to execute procedures for network attachment and communication setup. These procedures may take up to around ten seconds. The sensor nodes consume a few hundred milliwatt in average during these intervals, as well as during data transmission. Therefore, MNO UANs are much more energy-demanding for sensor nodes than the rest of UANs considered in this paper.

In this UAN class, the same device acts as both the sensor node and the gateway, which communicates with an MNO base station. The sensor node may use IP-based communication over the cellular link (Figure 3d)). At the application layer, CoAP is a more lightweight solution than HTTP. However, other data transports such as SMS may be used [27].

#### Emerging and Future MNO UANs

The high momentum of the Internet of Things (IoT), and its tremendous growth expectations with forecasts predicting up to 50 billion connected devices by 2020 [28], have significantly impacted on the deployment and/or design of 4G/5G technology.

Cellular MNOs and researchers have investigated how Long Term Evolution (LTE) can efficiently support M2M, also known as Machine-Type Communication (MTC) (MTC can be considered a synonym for M2M. which is very popular in the 4G context). It has been shown that, in comparison with other 2G/3G cellular network technologies, LTE (Release 8) provides a similar link budget, and greater capacity, while several proposals for device cost reduction for MTC have been evaluated [29].

On the other hand, LTE-Advanced (Release 10) was designed with particular consideration to MTC, including specific architectural components for MTC communications. Since the number of MTC devices in a cell is expected to be very large, access overload control mechanisms have been proposed [30]. Further work carried out by the 3GPP (Release 12) comprises solutions for efficient communication of small amounts of data (in terms of signaling overhead and MTC device power consumption) [31,32], reducing MTC device costs to be competitive with GPRS terminals targeting the same market [26], and overload control techniques which are demonstrated to achieve access success probability of almost 100% and average access delay in the order of 30 ms for 5000 MTC devices per cell [33]. A good summary of recent advancements in M2M communications in 4G networks can be found in [34].

Beyond 4G networks, the concern for MTC is even more fundamental. For example, *Massive machine communication* has been envisioned as one of the key horizontal topics in the METIS project, a flagship EU effort to define 5G networks. This project presumes a requirement to provide connectivity for 300,000 devices within one cell, enable long battery life (on the order of a decade) and low cost device implementations [35].

### 4.4. SO UANs

SO UANs constitute a very recent M2M solution gaining momentum in the urban scenario. In this type of UAN, SOs, which constitute a new category of cellular network operators, deploy their own base station infrastructure dedicated to sensor node connectivity. In contrast with traditional mobile networks, which were originally designed for voice applications and have evolved towards broadband services, SO networks are optimized for low throughput and low energy applications. Examples of SOs include SIGFOX and LoRa [36,37].

In SO UANs, sensor nodes communicate with base stations or gateways by means of a backhaul that consists of a very low bandwidth (in the range between tens and thousands of bit/s), long-range link (up to tens of kms), generally using a sub-GHz ISM band. This approach allows to cover a million-inhabitant city with a reduced number of base stations (e.g., in the order of three [36]). Base stations or gateways are connected by means of core networks to cloud servers which act as communication endpoints. The first deployments of SO UANs are being done without support for bidirectional communication. Whereas the protocols used in SO UANs are currently proprietary, a few SOs are pushing standardization efforts in organizations such as ETSI.

As in the previous UAN classes, in SO UANs, the radio of the sensor nodes is duty-cycled, with a sleep mode power consumption generally in the order of a few microwatts, and an active state power consumption around one hundred milliwatts.

### 4.5. DTN UANs

Finally, DTN UANs constitute a UAN class currently in experimental status [15]. DTN UANs exploit urban vehicles, such as public transportation buses or garbage trucks, equipped with a gateway that collects the data obtained by sensor nodes. Communication between the sensor nodes and the gateway occurs only when the gateway is within the coverage range of the sensor nodes and vice versa. The gateway on the vehicle can subsequently transmit the data, typically by means of a cellular connection. The temporary and infrequent connectivity between the sensor nodes and the gateway is a characteristic of DTNs, and the vehicle with the gateway plays the role of a *data mule* [38].

When sensor nodes are not mains-powered, the DTN UAN can only be feasible in terms of energy consumption if the radio of the sensor nodes is in sleep state by default. In order to efficiently collect data in such conditions, a radio-triggered wake-up system is used [39]. In this scheme, both the vehicle and the sensor nodes are equipped with two radio interfaces. The primary interface is a common wireless, low-power interface for data communication (e.g., IEEE 802.15.4). The secondary interface is a component of the wake-up system. At the vehicle side, the secondary interface transmits a special radio signal called wake-up signal. At the sensor node side, the secondary interface is a receiver designed to detect the wake-up signal. Although the wake-up signal receiver is always active, it consumes only a few microwatt. Upon detection of the wake-up signal, a sensor node activates its primary radio for data communication, otherwise it remains in sleep mode. The vehicle can thus collect the data from sensor nodes only when they are in its vicinity.

Connectivity of sensor nodes with the data mule only happens for reduced time periods which depend on the speed and/or stop time of the vehicle, and may have a duration of up to tens of seconds in the best case. On the other hand, the wake-up range that can be achieved with current systems is typically of around 30 m, although it can be increased by using directive antennas [40]. Furthermore, regulations may impose duty cycle constraints in the frequency bands used for the wake-up signals. Therefore, the connectivity time and the volume of data that can be exchanged is limited.

Note that whereas garbage trucks may typically delay data collection up to one day, they provide wide coverage since their routes cover the whole city. On the other hand, public transportation buses offer greater data collection frequency, but may cover a smaller area of the city.

Whereas the IRTF has produced protocol specifications for DTNs [41], these are overwhelmingly complex for constrained node networks. DTN UANs use more simple communication mechanisms. In order to enable communication between the gateway and the sensor nodes, IEEE 802.15.4 is generally used as the radio link technology. The data collection application operates on top of the link layer (Figure 3e). The gateway implements an IP-based protocol stack on top of the cellular link. Nevertheless, solutions for DTN UANs have not yet been standardized.

## 5. Discussion

This section discusses the characteristics of the presented UAN classes, examines them on the basis of the requirements derived from Smart City applications, and evaluates them in terms of deployment and sensor node coverage, latency, sensor node power consumption, standardization status, and economic cost. Table 2 provides a comparison of the main features of the considered UAN classes.

### 5.1. Deployment and Sensor Node Coverage

UAN sensor nodes need to be deployed in order to enable Smart City applications. However, UAN classes differ in the need and strategy for the deployment of infrastructure components (*i.e.*, backhaul and gateway). We next compare the UAN classes in this regard, and also point out the related implications in terms of sensor node coverage.

In LR-WPAN UANs, infrastructure has to be expressly deployed, which incurs installation and maintenance cost. However, the deployment can be optimized for providing coverage to sensor nodes located in specific points of interest for the intended applications.

In contrast, WLAN UANs avoid the need for an express deployment of backhaul and gateway nodes. Nevertheless, they are limited to the fact that WLAN infrastructure has been designed prior to the deployment of sensor nodes. Thus, they may not provide optimized (or even sufficient) coverage to all the sensor node locations.

On the other hand, MNO UANs allow, especially when 2.5G is used, the deployment of sensor nodes almost without geographic constraints, whereas SO UANs coverage is not currently the same, although their worldwide deployment is underway. In MNO or SO UANs, infrastructure is provided by the operator. However, when a sensor node is deployed, care must be taken to assure that the link between the sensor node (in its intended location) and the corresponding base station has sufficient quality. The flexibility in this regard is limited since the cellular infrastructure is typically managed by a third party.

Finally, DTN UANs do not require the deployment of fixed infrastructure throughout the city. Instead, a relatively low number of vehicles have to be provided with wake-up and gateway functionality. Of course, sensor nodes must be located close enough to data mule routes.

### 5.2. Sensor Node Power Consumption *versus* Notification Periodicity

**Figure 4:**
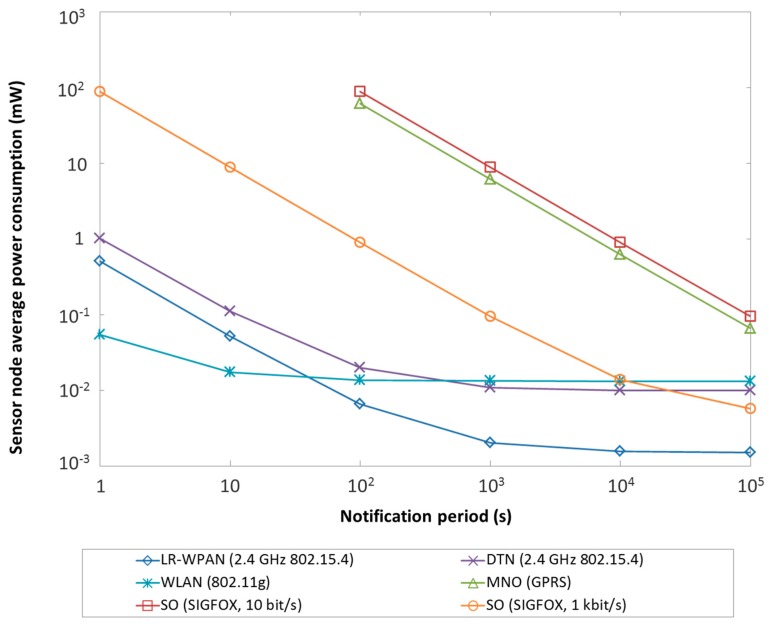
Sensor node average power consumption for representative enabling technologies of each UAN class, assuming periodic notifications. Note that the General Packet Radio Service (GPRS) MNO and the SIGFOX SO lowest data rate require notification periods greater than 10 s (see Figure 5).

A crucial performance parameter with deep implications in service availability and maintenance cost for a UAN class is sensor node average power consumption. Figure 4 illustrates this parameter for a representative module implementing an enabling technology of each type of UAN class, as a function of the sensor node notification periodicity.

**Table 2:** Feature comparison of the main UAN classes. Each individual column is related with the corresponding set of protocol stacks shown in Figure 3. This table summarizes content from Section 4 and Section 5.

		LR-WPAN (Figure 3a,b)	WLAN (Figure 3c)	MNO (Figure 3d)	SO	DTN (Figure 3e)
**Backhaul and gateway**	**Backhaul expressly deployed**	Yes	No	No	No	Yes (intermittent)
	**Gateway expressly deployed**	Yes	No (deployed a priori)	Yes (the sensor node includes the gateway)	No (deployed by the SIMless operator)	Yes (in public vehicles)
	**Can be extended/tuned by the municipality**	Yes	Yes	No (only the mobile operator can)	No (only the SIMless operator can)	Limited to available public vehicles
	**Power solution**	Mains power or batteries connected to streetlights	Mains power	Batteries or energy harvesting (gateway and sensor node implemented in the same device). Mains power desirable	Mains power	Gateway connected to vehicle battery
**Network characteristics**	**Latency (from sensor node to gateway)**	Milliseconds (per hop)	<Milliseconds (per hop)	Tens of seconds	Hundreds of milliseconds to tens of seconds	Minutes or hours
	**Latency (from gateway to sensor nodes)**	Minutes (Duty cycle period)	Minutes (Duty cycle period)	Minutes or hours (Duty cycle period)	Minutes or hours (Duty cycle period)	Minutes or hours (Time between connectivity events)
	**Permanent connectivity (sensor nodes point of view)**	Yes	Yes	Yes	Yes	No
	**Sensor node location degree of freedom supported by the UAN**	High	Medium	Medium/High	Medium	Low
**Smart City application support**	**Event-based applications**	Yes	Yes	Yes (latency has to be considered)	Yes (latency has to be considered)	No
	**Notification periodicity (based on sensor node power consumption)**	>10 s	> 10 s	>1 h	≥10 min (at 1 kbit/s) > 1 h (at 10 bit/s)	1 h to 1 day (due to connectivity limitations)
**Additional services**		No	Video, web access	Image transfer, web access	No	No
**Standardized communication protocols**		Yes	Yes	Yes	No (in progress)	No

**Figure 5:**
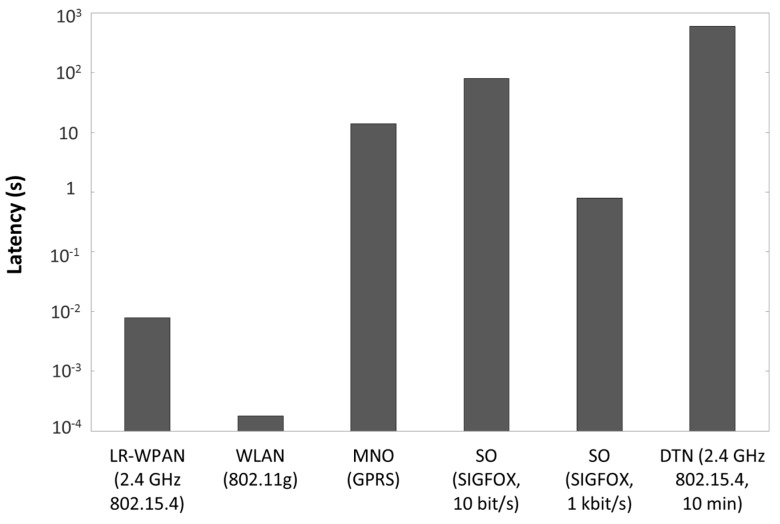
Latency of data transmitted by sensor nodes to their next hop for representative enabling technologies of each UAN class.

In this study, the sensor node is assumed to transmit a data unit in acknowledged mode (except for the SIGFOX SO UAN, whereby a unidirectional communication mode is offered, leveraging that several base stations participate in signal reception) every notification period, and remain in sleep state otherwise. Average sensor node power consumption results were theoretically calculated on the basis of a power consumption characterization (in terms of current consumption and duration) of each state involved in the cycle that comprises the notification transmission and the sleep period, for each module considered. For the LR-WPAN UAN, the power consumption characterization was obtained from published empirical measurements of the CC2430 2.4 GHz IEEE 802.15.4 platform [42]. For the MNO UAN, we derived the power consumption characterization by performing measurements of the WISMO 228 GPRS radio platform [43], by using a N6750 DC Power Analyzer. For the DTN UAN, we used the same power analysis tool to model the radio-triggered wake-up receiver presented in [40]. For the WLAN and SO UANs, we characterized the power consumption of the RN-171 module in IEEE 802.11g mode and the TD1202 SIGFOX module, respectively, on the basis of information reported in their datasheets [24,44]. Typical transmit power settings for each technology have been considered, *i.e.*, 0 dBm for LR-WPAN, DTN and WLAN, 10 dBm for SIGFOX and 33 dBm for GPRS. An equivalent message payload size of 100 bytes has been assumed (although SIGFOX limits the payload size to 12 bytes). This payload size is in the order of magnitude of the IEEE 802.15.4 frame maximum payload size, and therefore it represents a reference on the maximum expected size of messages used in sensor node applications (note that, if shorter messages are actually transmitted, sensor node power consumption will actually be lower than the one depicted in Figure 4 for any UAN class). For the GPRS study, a microcontroller consuming 1 μA in sleep mode has been assumed.

As shown in Figure 4, sensor node average power consumption is asymptotically dominated by the sensor node current consumption in sleep mode. LR-WPAN, with submicroampere sleep mode current consumption, achieves the best performance for a notification period beyond one minute. WLAN modules exploit their high bitrate, which compensates their high transmit power consumption, to provide the best performance for very frequent data communication (which however is not characteristic of Smart City applications). DTN sensor nodes permanently consume additional power compared with LR-WPAN platforms to feed the wake-up receiver, however they achieve an asymptotic behavior similar to that of WLAN modules. Sensor nodes that use a GPRS MNO interface are penalized due to the power consumed in actual communication, and only allow low-power operation for notification periods in the order of tens of hours. SIGFOX ultranarrowband 10 bit/s channels suffer a similar problem, whereas use of the SIGFOX 1 kbit/s rate reduces power consumption due to a lower transmit time, and achieves better asymptotic performance than WLAN or DTN solutions thanks to a lower sleep mode power consumption.

### 5.3. Latency and Event-Based Application Support

Latency is one of the most critical performance parameters of a UAN, since it determines whether the UAN can support event-based applications. Figure 5 depicts the latency of a 100-byte payload data unit transmitted by sensor nodes to their next hop, for a representative module implementing an enabling technology of each type of UAN class. For the MNO UAN, the result is obtained from the empirical measurements mentioned in the previous subsection. For the rest of UANs, latency is calculated theoretically, assuming an error-free scenario, and including the acknowledgment delay for LR-WPAN and WLAN UANs. For the DTN UAN, a period between connectivity opportunities of 10 min has been assumed for comparison purposes. This period has been chosen as an optimistic value, considering a relatively frequent rate of connectivity opportunities, which may be found when public buses play the role of data mules. Note that the period between connectivity opportunities may be even in the order of a day, when garbage collection vehicles are used as data mules. Latency in DTN UANs is strongly dominated by the period between connectivity opportunities, whereas in the rest of UAN classes, connectivity is permanent.

As shown in Figure 5, in LR-WPAN and WLAN UANs, the latency of data transmitted by sensor nodes is low (*i.e.*, up to a few milliseconds per hop). SO UANs may lead to delays between hundreds of milliseconds up to tens of seconds, depending on the bit rate used. MNO UANs offer delays up to around ten seconds, which do not allow real-time transmissions, but are sufficient for event-based applications in Smart Cities. In DTN UANs, sensor nodes have to wait for minutes or hours until the next connection opportunity with a data mule for data communication. Therefore, DTN UANs constitute the only UAN class that does not support event-based applications.

In the opposite direction, the sensor node duty cycle (or the connectivity opportunity rate in DTN UANs) determines the delay until data can be sent to the sensor node from the previous hop, which can typically be in the order of minutes, or even hours.

### 5.4. Communication Protocols Standardization Status

A networking paradigm can only reach a wide community if it is based on (*de facto* or *de jure*) standard protocols. LR-WPAN, WLAN and MNO UANs use open and standard protocols at all layers (note that the development of certified ZigBee products for commercial purposes requires payment of a fee). Remarkably, in IP-based LR-WPAN, WLAN and MNO UANs, the sensor nodes use IP, thus contributing to the Internet of Things (IoT). A key advantage of supporting IP is effortless Internet connectivity, and scalable application development, independent of the specific layers below IP. On the other hand, SOs are currently contributing to standardizing SO UAN protocols. Finally, communication protocols for DTN UANs have not yet been standardized, and constitute currently an open issue.

### 5.5. Economic Cost

We next estimate the economic cost of each UAN class in an example scenario, from the point of view of the entity responsible for UAN deployment and management (e.g., a municipality). We consider a 1 km^2^ urban area with a total of 1000 sensor nodes. This sensor node density matches the characteristics of a 30,000-inhabitant city called Sant Vicenç dels Horts, whereby the first ever smart city pilot was deployed in Spain, to our best knowledge. In this city, the number of streetlights and garbage containers per km^2^ is approximately equal to 800 and 200, respectively. These numbers provide an order of magnitude on the sensor node density that can be expected in the type of municipalities (in the range between 20,000 and 50,000 inhabitants) that contribute the highest fraction to the total population of the country [45]. Note, however, that sensor node density and municipality characteristics may vary across cities.

On the other hand, for the economic cost calculations, we assume the pricing data shown in Table 3, based on current market costs. Device acquisition costs include microcontroller, network interface(s), transducers, and robust encapsulation costs for outdoor deployment. Installation costs comprise roadworks costs for all types of devices, whereas, in addition, the installation of backhaul nodes and gateways requires works for connecting these devices to the power grid. Note that gateways need a more robust physical support than backhaul nodes, due to the greater weight and form factor of the former, thus increasing their installation cost. The cost increase is greater when the gateway has to be deployed on the street (for LR-WPAN UANs) than in a vehicle (for DTN UANs).

**Table 3:** Pricing data in Euro used in estimating the economic cost of UANs. A yearly maintenance cost equal to 15% of the acquisition cost, and a battery replacement cost equal to the installation cost are assumed. A sensor node installation cost of 100 Euro is assumed. (a) WLAN backhaul has been deployed a priori; (b) In MNO UANs, the sensor node and the gateway are implemented in the same device; (c) The indicated SO subscription fee includes data web hosting services.

	Sensor Nodes			Backhaul Nodes			Gateways				
	Acq. Cost	Subsc. Fee	Battery Acq. ct.	Num-Ber	Acq. Cost	Instal. Cost	Num-Ber	Acq. Cost	Instal. Cost	Internet Fee	Elec. Fee
**LR-WPAN**	200	-	7	200	200	330	20	1000	1130	20	2.7/month
**WLAN**	200	-	14	(a)	(a)	(a)	-	-	-	-	-
**MNO (GPRS)**	300	1/month	20	-	-	-	(b)	(b)	(b)	(b)	-
**SO**	200	2/year (c) [36]	14	-	-	-	-	-	-	-	-
**DTN**	250	-	7	-	-	-	1	800	500	10/month	-

**Figure 6:**
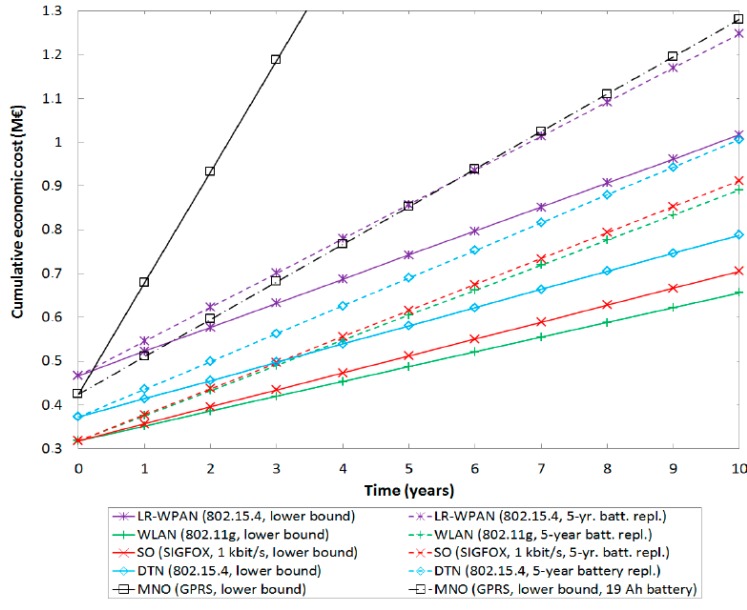
Economic cost estimate of each UAN class, based on Table 3 pricing data.

Figure 6 illustrates the cumulative economic cost of each UAN class as a function of time, considering the initial deployment cost, as well as averaged yearly operation, maintenance and consumption (OMC) costs. The deployment cost includes acquisition and installation of sensor nodes, backhaul nodes and gateways (when necessary), whereas OMC costs include device operator subscription fees, infrastructure mains power consumption, and maintenance including sensor node battery replacement. For the latter, two economic cost values are considered: a lower bound on the battery replacement cost, which corresponds to the lifetime of an ideal 2.2 Ah AA-category battery under a regime of sending 1 notification/h, and an example scenario where such batteries have to be replaced every 5 years. AA-category batteries offer a good trade-off between cost and capacity, being 2.2 Ah a typical capacity value for this type of batteries. Note that battery characteristics will determine actual battery replacement frequency and cost. The rate of 1 notification/h has been assumed as a canonical value, intended to capture the order of magnitude of the average notification rate in Smart City applications (see Table 1).

LR-WPAN UAN requires the greatest initial investment, since in addition to the sensor nodes, and in contrast with the rest of UAN solutions, backhaul nodes and gateways have to be expressly deployed. The initial investment in DTN UANs is greater than that of SO and WLAN UANs mainly because DTN UAN nodes are slightly more expensive due to the use of radio-triggered wake-up receivers. OMC cost lower bounds (*i.e.*, the slope in the corresponding curves in Figure 6) are similar across technologies, since they strongly depend on the number of sensor nodes. In LR-WPAN UANs, the additional maintenance cost of infrastructure (*i.e.*, backhaul nodes and gateways) is compensated by the low power consumption of sensor nodes, which leads to a low battery replacement frequency. Note that LR-WPAN UAN infrastructure would be more expensive in relative terms in scenarios of lower sensor node density. Finally, in MNO UANs the high power consumption leads to an exacerbated battery replacement cost, which can be mitigated by exploiting greater capacity batteries (see the example of using 19 Ah batteries in Figure 6). Nevertheless, greater OMC costs penalize MNO UANs in the long term.

Remarkably, the economic cost of storing the data collected by UANs is significantly lower than the UAN economic cost. Cloud storage and computing has been identified as a vital technology for data storage and processing in the Smart City [6,46,47]. Cloud servers commonly make the biggest contribution to cloud hosting infrastructure costs. Cloud providers offer cloud server resources such as RAM, storage capacity, CPU power, and the supporting operating system. Current cloud provider pricing schemes depend on the amount of resources to be used by the customer, and may be in the order of 50€–1000€ per year for 10–200 GB of storage [48]. As a reference, 1000 sensor nodes transmitting individually a 100-byte payload every hour produce a total amount of data (which has to be enriched with metadata for useful information analysis) greater than 1 GB/year. Note that other services such as camera surveillance produce notably greater data volumes. Future Smart City cloud data storage and computing systems may be complemented by the emerging Fog computing paradigm [49].

## 6. Mobile Sensing Networks in the Smart City

As it has been presented in previous sections, UANs comprise fixed sensor and actuator nodes. However, there exists a family of emerging networking paradigms that exploit sensing, computing and communication resources available on mobile devices, such as personal consumer electronics equipment (most notably, smartphones) or in-vehicle systems, for data collection and processing [50,51]. We refer to this category of networks as Mobile Sensing Networks (MSNs).

MSNs can complement and cooperate with UANs to enable Smart City applications [52]. UANs provide the solution for the subset of Smart City applications that require the deployment of fixed sensor nodes in specific locations, such as garbage container occupancy sensors, underground humidity sensors in green zones, leak/breakdown sensors in utility infrastructure or security sensors in restricted access zones. Otherwise, MSNs may enhance UAN operation, by providing additional sensed data. However, MSNs alone cannot offer reliable and predictable data collection, as discussed in subsequent paragraphs. Table 4 shows the Smart City applications enabled by UANs or MSNs.

**Table 4:** Smart City applications enabled by UANs or MSNs. Note that MSNs alone cannot offer reliable and predictable data collection. The list of applications is the same as the one in Table 1.

	UANs	MSNs
**Garbage collection**	Yes	No
**Lighting control**	Yes	Yes
**Green zone management**	Yes	No
**Environmental control**	Yes	Yes
**Parking availability**	Yes	Yes
**Street traffic**	Yes	Yes
**Utility infrastructure**	Yes	No
**Security**	Yes	No

In comparison to UANs, MSNs exhibit a few significant advantages. MSN devices are less resource-constrained than the usual mote-type of UAN sensor nodes. On the other hand, billions of MSN units (either mobile devices or connected vehicles) are already *deployed*, *i.e.*, are located in close proximity of their human users, which may reduce fixed sensor node deployment costs [50]. However, MSNs introduce additional challenges and complexity. Predicting the resources (e.g., energy or bandwidth) that may be needed to carry out a given task, and even whether the task itself can be performed, is difficult. Further, challenges arise in dense scenarios, due to wireless bandwidth limitations, which require techniques for efficient channel utilization [51]. Finally, MSNs rely on the willingness of the users to contribute to data collection, and require more complex processing operations to mitigate the intrinsic low reliability of the collected data (e.g., a smartphone measuring light levels might be temporarily in the user’s pocket).

The devices of a MSN may use radio interfaces such as IEEE 802.11 variants or cellular (e.g., 3G/4G) to communicate between themselves or to transmit data via the Internet to a Smart City management center, where the data can be processed and stored. Data obtained by both UANs and MSNs can potentially be combined in the management center to enrich the overall data collection, decision and actuation process.

We divide MSNs in smartphone-centric MSNs and vehicle-centric MSNs (the latter are also known as Vehicular Sensor Networks, VSNs). The next two subsections focus on these two MSN classes, respectively.

### 6.1. Smartphone-Centric MSNs

Smartphone-centric MSNs are mainly composed of smartphones (although they may comprise other consumer electronics devices such as music players, wearable devices, *etc.*), which may or may not communicate with each other, that use their available sensors to obtain information from their environment. This type of MSNs allow a paradigm called mobile crowdsensing, by which phenomena are monitored by a community of observers [50]. This paradigm has high potential in the Smart City [52,53].

A smartphone can generally provide information about temperature, atmospheric pressure, light intensity, GPS location, acceleration, gyro or magnetic compass, among others. Further information can be derived by processing the available sensed data samples, such as, e.g., the physical activity of the citizen or the traffic congestion while driving.

Mobile crowdsensing is intrinsically dynamic (with user mobility in the range of pedestrian to vehicular speeds), and poses predictability issues. On the other hand, efficient operation requires the use of collaborative sensing techniques. To this end, distributed architectures and policies have been developed [52].

### 6.2. Vehicle-Centric MSNs

These networks comprise smart vehicles—equipped with on-board sensors—that can communicate with each other, and thus form a Vehicular Ad-Hoc Network (VANET), or with road side infrastructure, to enable a variety of road monitoring, driving safety, emergency response, and parking availability applications [50,54,55].

One of the basic issues in vehicle-centric MSNs is achieving an efficient use of the wireless medium, which is prone to become congested in dense scenarios due to the transmission of data readings from vehicles. Solutions have been proposed mainly in two areas: (i) mechanisms for fair shairing of the available bandwidth; and (ii) reduction of the transmitted data by exploiting correlation in time and space of the observed physical magnitudes and/or events [51]. Other problems of vehicle-centric MSNs comprise high mobility of the network nodes, as well as network partitioning. Researchers have developed good performing solutions for these scenarios, using DTN-inspired concepts, whereby otherwise traditional sensor network protocols would fail [54,56].

## 7. Smart City Modeling: Related Work

This manuscript has offered a UAN modeling and evaluation framework. In this section we review literature work in two main areas relevant to this manuscript: (i) Smart City conceptual modeling; and (ii) technical ICT Smart City component modeling.

Several works have attempted to model the Smart City concept from a comprehensive perspective [12,57,58,59]. Authors in [12] identify the following eight elementary components which represent a smart city framework: management and organization, technology, governance, policy context, people and communities, economy, built infrastructure, and natural environment. Other researchers state that there exist six Smart City dimensions agreed by the scholar community, namely: people, government, economy, mobility, environment and living [57]. Another work defines that a city is *smart* when investment in human and social capital, transport and ICT infrastructure produces economic and life quality benefits, while making an efficient use of resources and involving citizen participation in the city government [59]. A recent work introduces the Sensing as a Service (SaaS) model for the Smart City, highlighting the convergence of the IoT and Smart City spaces, while recognizing the aforementioned six Smart City dimensions [34]. Authors in [13] provide a methodology based on the definition of use cases, which can be expanded by so called integration profiles, which provides a systematic way to model the elements involved in a Smart City and their interactions. For example, the UAN model described in our paper could be structured following a similar approach.

Regarding the technical ICT components that enable the Smart City, generic architectures for supporting heterogeneous Smart City applications have been devised [3,7,60]. Such architectures comprise sensor/actuator networks, connectivity means, big data processing and storage platforms, as well as application interfaces. Focusing on the sensor/actuator subsystem, researchers have aimed attention at how sensing technology is applied to enable various Smart City applications [6], as well as on networking aspects, such as the requirements for routing in low-power and lossy networks in urban environments, although the different approaches that emanate from different UAN classes have not been considered [4]. Several works present the development of and/or provide experimental results from Smart City testbeds and pilot projects in various cities such as Santander, Padova, Barcelona, Beijing and Oulu [3,5,8,9,10,11]. Remarkably, the architecture of the 20,000-IoT-device test facility developed in the SmartSantander EU project is described in [3]. A more recent work introduces the IoT-based Smart City architecture deployed in Padova [5]. The main focus in these works is demonstrating the feasibility of Smart City concepts by means of sensor network deployments.

We conclude from the literature review that while significant efforts have been devoted to modeling the Smart City from a conceptual point of view, and even though technical descriptions of the ICT infrastructure that supports Smart Cities exist as well, to our best knowledge, a comprehensive UAN model and evaluation such as the one presented in this paper has not been published as of the writing.

## 8. Conclusions

This article has introduced the concept, requirements and architecture of UANs, and has examined the main current and emerging UAN classes. From the study, we conclude that LR-WPAN UANs enable the widest range of Smart City applications, at the expense of incurring a high economic cost. Whereas WLAN UANs are promising, they exhibit coverage and flexibility limitations due to the reuse of priorly deployed infrastructure. On the other hand, some SO UAN variants and DTN UANs efficiently support applications that involve sporadic transmissions. The latter require adequate data mule route planning and, on the other hand, constitute the only UAN class not suitable for event-based applications. Finally, MNO UANs offer good sensor node location flexibility, but should only be used for infrequent transmission applications, and show a high long-term economic cost.
